# Electromyographic biofeedback-assisted pelvic floor muscle training for female stress urinary incontinence: a systematic review and meta-analysis

**DOI:** 10.3389/fmed.2026.1789923

**Published:** 2026-06-29

**Authors:** Jie Liu, Yu Liu, Shen Liu, Jingwen Chen

**Affiliations:** 1 Ganzhou Cancer Hospital, Gannan Medical University, Ganzhou, Jiangxi, China; 2 The First Affiliated Hospital of Gannan Medical University, Ganzhou, Jiangxi, China; 3 Ganzhou Hospital-Nanfang Hospital, Southern Medical University, Ganzhou, Jiangxi, China

**Keywords:** electromyographic biofeedback, meta-analysis, pelvic floor muscle training, stress urinary incontinence, women health

## Abstract

**Background:**

Pelvic floor muscle training (PFMT) is the preferred initial therapy for female stress urinary incontinence (SUI); however, achieving proper muscle activation and sustaining adherence over time are often difficult. Electromyographic biofeedback (EMG-BF) may facilitate motor learning during PFMT; however, its added benefit over PFMT alone remains uncertain.

**Objective:**

We investigated the clinical effects of EMG-BF-supported PFMT vs. standard PFMT in women with SUI.

**Methods:**

This systematic review and meta-analysis followed PRISMA 2020 and a prospectively registered protocol (PROSPERO: CRD420261281430). PubMed, Embase, Web of Science, Cochrane Library, and Scopus were searched from inception to 9 January 2026. RCTs enrolling adult women with SUI and directly comparing EMG-BF-assisted PFMT vs. PFMT alone were included. Outcomes were pooled as standardized mean differences (SMDs) with 95% confidence intervals (CIs). Risk of bias was assessed using Risk of Bias 2 (RoB 2). Heterogeneity was quantified using *I*^2^, and small-study effects were explored via funnel plots and Egger's test when appropriate.

**Results:**

Eight RCTs (*n* = 1,045; EMGBF + PFMT: 518; PFMT: 527) were included. Compared with PFMT alone, EMG-BF-assisted PFMT yielded a small reduction of incontinence severity (*SMD* = −0.17, 95% *CI* −0.30 to −0.05; *I*^2^ = 0%) and mild benefit of QoL (*SMD* = −0.21, 95% *CI* −0.34 to −0.08; *I*^2^ = 35%). PFMS was improved moderately (*SMD* = 0.56, 95% *CI*: 0.09–1.03; *I*^2^ = 51%). The leave-one-out sensitivity analysis on UISS did not change the results. Egger's test suggested potential small-study effects for incontinence severity (*p* = 0.0118).

**Conclusions:**

The addition of EMG-BF to PFMT provides small but statistically significant improvements in symptoms and quality of life (QoL) compared with PFMT alone, with a greater benefit observed for pelvic floor muscle strength. This supports the use of select, rather than regular, use of EMG-BF-particularly in those who have difficulty recognizing the correct contraction of the PF, and/or need extra structure at the beginning.

## Introduction

Stress urinary incontinence (SUI) is a common type of urinary incontinence in women, defined by involuntary leakage during activities that raise intra-abdominal pressure (e.g., coughing, sneezing, or physical exertion) (1). In addition to being bothersome, SUI is associated with limitations in activities of daily living, reduced social participation, and psychological distress. Psychological factors, such as anxiety and stress are often reported amongst women suffering from UI and may play a role in interaction with the perception of symptoms and seeking of help (2). SUI also impacts on intimacy and sexual wellbeing, and observational evidence shows associations of PFM functioning with measures of sexual function in women with or without SUI (3), and more general reviews show that incontinence may affect quality of life (QoL), mental health and sexual activity (4). Such effects offer an obvious justification for focusing on conservative, treatable symptoms for which there are effective treatments to improve both symptom severity and patient-reported outcomes.

SUI has a multifactorial etiology involving anatomical, neuromuscular, hormonal and lifestyle factors. The post-partum state represents one of the most common clinical settings for the development of SUI, and pelvic floor dysfunction can occur alongside of other musculoskeletal changes such as diastasis rectus abdominis (5). Imaging-based work has further described pelvic floor parameters that predict postpartum SUI, emphasizing the contribution of pelvic support structures and urethral function to continence mechanisms (6). Obesity is another well-recognized contributor, with narrative work summarizing how obesity-associated changes may affect pelvic floor tissues, neuromuscular integrity, and continence physiology (7). Hormones are also likely to be clinically relevant; pelvic floor muscles express hormone receptors and it is therefore plausible that endocrine change throughout pregnancy, menopause, and aging can modulate pelvic floor structure and function and alter vulnerability for pelvic floor disorders (8). Moreover, some patients' subpopulations might be subject to increased load of pelvic floor symptoms that could complicate the treatment process. For instance, women with connective tissue disorders including Ehlers–Danlos syndrome describe significant symptoms of the pelvic floor (9), and neurologic disease (e.g., multiple sclerosis) could pose even more of a challenge to conservative care options and continued training (10). Taken together, these data further support that SUI is prevalent, clinically heterogenous, and poised to grow its burden within health care systems. This also increases the need for identifying the best, most scalable conservative policies.

PFMT is widely accepted as a first-line conservative treatment for women with urinary incontinence and is supported by high-quality meta-analyses (11, 12). Its mechanism is biologically plausible, as improvements in pelvic floor muscle strength, endurance, and coordination may enhance urethral support and closure during increases in intra-abdominal pressure (13). Yet, the success of PFMTs in practice requires appropriate activation of these muscles as well as compliance with a regular programme. Many women have difficulty voluntarily activating the pelvic floor muscles and performing an isolated contraction without guidance, and incorrect technique may reduce the effective training dose (14). Home-based interventions face the same issue with adherence as other types of physical activity interventions, and pragmatic evaluations of home based adherence show that long-term compliance can be elusive in the absence of motivational techniques. In particular, even if the power increases, it does not necessarily mean that the symptoms will reduce proportionally. Mechanistic synthesis suggests that PFMT causes morphological and functional change; however the relationship between changes in the variables of the pelvic floor and symptoms outcome may differ, suggesting that the development of continence is a process involving much more than just increasing strength (15). Such issues motivate adjunct approaches to improve the quality of training, supporting skill learning, and keeping compliance long term.

Electromyographic biofeedback (EMG-BF) was proposed as an aid to PFMT in order to overcome some of the technique and adherence barriers which might limit the success of PFMT alone. EMGBF shows pelvic floor myoelectric activity, usually through the use of intravaginal probes or perineal surface electrodes, providing instant visual or auditory feedback which may assist a woman with confirming she is contracting and relaxing correctly. Our idea relies on the concept of motor learning: external feedback could enhance perception about contraction quality, and might lead to neuromuscular re-education, especially at the beginning of learning a task. Empirical data from other fields than PF rehabilitation further support that feedback manipulation may alter estimations as well as execution of MVC, which is in line with the idea of feedback shaping motor output (16). In the context of pelvic floor disorders, bio-assisted PFMT have been related to improvement on pelvic floor functioning in women with pelvic floor dysfunction, supporting the plausibility of using feedback to improve training quality (17). With further improvement in electrophysiological acquisition technology (e.g., better electrode designs), there is also interest in optimizing signal quality, comfortability and practicability of the EMG based monitoring (18). However, the clinical adoption is also affected by availability, clinician training, as well as patient's acceptance (due to many patients being uncomfortable or reluctant in using an invasive device) (19).

Although there is an obvious theory-based reason for this, it has been questioned whether the added value of EMGBF over good PFMT performance is worth pursuing or not. Reviews pooling heterogeneous population and interventions showed inconclusive results for instance, the conclusions of a systematic review comparing PFMT alone vs. with an adjunct including biofeedback/electrostimulation found that PFMT is effective in treating UI although it was inconclusive about adjunct superiority by severity of symptoms; however, wide ranging inclusion criteria and mixed intervention class makes it difficult to attribute the effects to EMG-BF in particular (20). Likewise, the inclusion of mixed diagnostic categories with varied study designs was also evident in a meta-analysis reporting benefits of EMGBF across multiple outcomes, which can inflate heterogeneity, and restricts causal inference on SUI specific questions (21). A key methodological issue is that “biofeedback arms” will have greater therapist contact, more supervised sessions or higher exercise dose, and observed improvements might be attributable to the training exposure as opposed to feedback *per se*.

A major attempt to resolve this question comes from the OPAL multicentre randomized controlled trial. OPAL was designed to standardize PFMT content across groups and isolate the effect of adding EMG-BF. The trial reported that PFMT plus EMG-BF was not superior to PFMT alone for urinary incontinence severity and related outcomes at longer-term follow-up, leading the authors to discourage routine use of EMG-BF as an adjunct in typical care pathways (22). At the same time, OPAL excluded women unable to contract the pelvic floor on examination, leaving uncertainty as to whether a subgroup with greater skill acquisition needs might experience more benefit from EMG-BF (22). Therefore, an important unresolved question is not simply whether EMG-BF “works,” but under what circumstances—patient phenotype, baseline contraction ability, PFMT dose and supervision, and outcome domain—it adds measurable value beyond PFMT.

Separately, changing paradigms around how PFMTs are delivered offer some interpretive framework for the adjunct effect. Telehealth platforms and virtual coaching strategies can increase engagement with training sessions, offering a different route to expanding access to conservative management. In real-life settings, telemedicine solutions for women's stress urinary incontinence have shown improved symptoms following iterative patient feedback loops (22). App-guided PFMT has been shown in randomized trials to improve selfefficacy and outcome in postpartum populations (23), and smartphone reminders could potentially improve adherence and pelvic floor measures in the postpartum period (24). Models of teleconsultation are also acceptable to patients and may be useful for maintaining training access if face to face care is limited (25). In addition, economic evaluations suggest that group-based PFMT may represent value for money in older women, emphasizing that organization of service provision can have an important effect on feasibility and scalability (26). This suggests as a pragmatic matter that if the comparator is “good quality PFMT with good support”, the marginal gain of adding in EMGBF may not be as large if PFMT is being given under minimal supervision and little adherence support.

Therefore, our current systematic review and meta-analysis is restricted to women who have SUI and directly compares EMGBF-assisted PFMT vs. PFMT alone. We pool the data across clinically important endpoints—urinary incontinence (UI) severity, pelvic floor muscle strength, and QoL—and to contextualize results with respect to the amount of intervention dose, level of supervision, length of follow up, and possible small-study effects. This is the first study that combines data from small mechanistic trials with data from a large pragmatic trial aiming at answering which way of using EMGBF would be better: selective or routine use within conservative treatment of SUI.

## Methods

### Study design and registration

This meta-analysis was conducted in accordance with the PRISMA 2020 statement and prespecified methods in a prospectively registered protocol (PROSPERO: CRD420261281430).

### Eligibility criteria

**Design:** Randomized controlled trials (parallel-group or multi-arm RCTs).

**Population:** Adults diagnosed with SUI. Trials enrolling mixed UI were eligible only if SUI data were separately extractable or SUI was the predominant diagnosis according to the trial's definition.

**Intervention:** PFMT with EMG biofeedback (clinic-based and/or home-based EMG-BF), delivered as a structured training program.

**Comparator:** PFMT without EMG biofeedback (usual PFMT, supervised PFMT, or home PFMT), with comparable co-interventions where possible.

**Outcomes:** At least one of the following: Urinary incontinence severity (e.g., ICIQ-UI SF, ICIQ-SF, pad test, diary-based episodes); Pelvic floor muscle strength (e.g., perineometry, Modified Oxford scale); Condition-specific or generic QoL (e.g., IIQ-7, I-QOL, KHQ, EQ-5D);

Exclusion criteria included non-randomized studies, uncontrolled studies, studies without extractable outcome data, and trials where the intervention was not EMG-based biofeedback (e.g., pressure biofeedback only) unless EMG-BF effects could be isolated.

### Information sources and search strategy

We searched PubMed, Embase, Web of Science, the Cochrane Library, and Scopus from database inception to 9 January 2026. The search strategy combined controlled vocabulary (e.g., MeSH and Emtree terms) and free-text keywords covering the condition, intervention, and biofeedback modality, including terms related to SUI, PFMT, electromyography/EMG, and biofeedback. Where applicable, synonyms and spelling variants were used, and search strings were adapted to each database's indexing system and interface. In addition, we performed backward citation searching by screening reference lists of all included trials and relevant reviews to identify further eligible randomized controlled trials.

### Data extraction

Two reviewers (JL and JWC) independently extracted data using a predefined extraction form, including: study characteristics (year, country, setting, design), participant characteristics (diagnostic criteria, mean age, baseline severity), intervention and comparator details (training duration, frequency, supervised sessions, home component, EMG-BF device and parameters), outcomes, follow-up time points, and attrition. When multiple follow-up time points were available, outcomes were extracted at the end of intervention and at the longest available follow-up, consistent with the protocol.

### Risk of bias assessment

Risk of bias was assessed independently by two reviewers using the Cochrane Risk of Bias 2 (RoB 2) tool across domains (27): randomization process, deviations from intended interventions, missing outcome data, measurement of outcomes, and selection of reported results. Any disagreements were resolved by discussion.

### Data synthesis and statistical analysis

Meta-analyses were performed in Stata 18.0. For continuous outcomes measured using different instruments, we pooled effects using the standardized mean difference (SMD; Hedges' *g*) with 95% confidence intervals (CIs). For dichotomous outcomes (e.g., cure/improvement), we calculated risk ratios (RRs) with 95% *CI*s whenever event data permitted (odds ratios were used only when RRs could not be derived consistently across trials). Because clinical and methodological diversity across trials was anticipated (e.g., differences in PFMT dose and supervision, EMG-BF delivery mode, and outcome measures), our default, pre-specified approach was to use a random-effects model (DerSimonian–Laird). Statistical heterogeneity was assessed using Cochran's *Q*-test and quantified using *I*^2^ and τ^2^. In line with our prespecified decision rule, when heterogeneity was low (*I*^2^ ≤ 50%), we additionally examined fixed-effect estimates; otherwise, random-effects results were retained as the primary analysis. Planned sensitivity analyses included leave-one-out analyses for the primary outcome and analyses restricted to studies judged as low risk of bias. Small-study effects/publication bias were assessed only when ≥10 studies were available for an outcome, using funnel plots and Egger's regression test, with cautious interpretation given the limited power of these methods when few studies are included.

## Results

### Study selection

**Figure 1:**
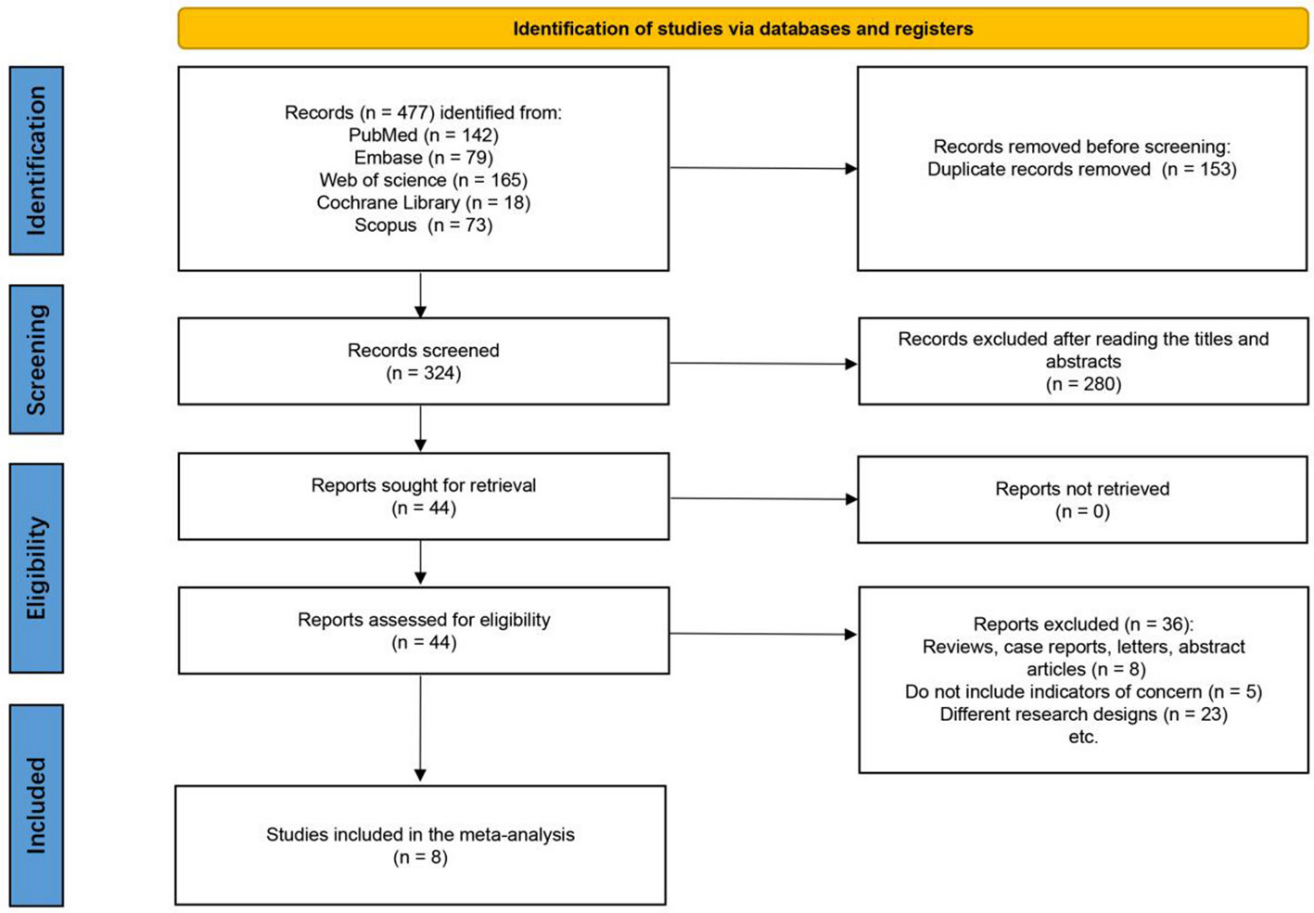
PRISMA flow diagram of the study selection process for the systematic review and meta-analysis of EMG-BF-assisted PFMT.

The search yielded 477 records (PubMed, *n* = 142; Embase, *n* = 79; Web of Science, *n* = 165; Cochrane Library, *n* = 18; Scopus, *n* = 73). After removal of 153 duplicates, 324 citations underwent title/abstract screening, and 280 were excluded. We assessed 44 full-text articles for eligibility; 36 were excluded (reviews/case reports/letters/abstract-only publications, *n* = 8; no relevant outcomes, *n* = 5; ineligible study design, *n* = 23). Eight RCTs (28–35) were ultimately included in the meta-analysis (Figure 1).

### Characteristics of included randomized controlled trials

The characteristics of the included randomized controlled trials are summarized in Table 1. A total of eight RCTs (28–35) were included. Trials were conducted in China (*n* = 2), Turkey (*n* = 2), Japan (*n* = 1), Brazil (*n* = 1), and the USA (*n* = 2, as reported in the included table). Sample sizes ranged from 31 to 600 participants, with intervention duration between 3 and 16 weeks, and follow-up ranging from 8 weeks to 24 months. Across all studies, the experimental intervention was EMG biofeedback-assisted PFMT, and the comparator was PFMT alone.

**Table 1:** Characteristics of included randomized controlled trials.

Study	Country	Study design	Intervention		Sample size (***n***)		Age (years)		Treatment time (w)	Follow-up time (m)	Outcomes
			E group	C group	E group	C group	E group	C group			
Goode (29)	USA	RCT	EMG-BF plus PFMT	PFMT	70	70	66.8 ± 7.0	66.3 ± 7.5	8 weeks	12 months	Severity of urinary incontinence, QOL, cure and improvement rate
Liu et al. (32)	China	RCT	EMG-BF plus PFMT	PFMT	58	60	50 ± 9.9	46 ± 11.7	12 weeks	12 weeks	Severity of urinary incontinence, QOL
Özlü et al. (33)	Turkey	RCT	EMG-BF plus PFMT	PFMT	17	17	42.11 ± 8.33	42.82 ± 6.30	8 weeks	8 weeks	Severity of urinary incontinence, QOL, pelvic floor muscle strength, cure and improvement rate
Wu et al. (35)	China	RCT	EMG-BF plus PFMT	PFMT	23	22	32.2 ± 4.7	32 ± 5.2	6 weeks	6 months	Severity of urinary incontinence, QOL
Bertotto et al. (28)	Brazil	RCT	EMG-BF plus PFMT	PFMT	16	15	58.4 ± 6.8	59.3 ± 4.9	4 weeks	8 weeks	Severity of urinary incontinence, pelvic floor muscle strength
Hagen et al. (30)	USA	RCT	EMG-BF plus PFMT	PFMT	300	300	48.2 ± 11.6	47.3 ± 11.4	16 weeks	24 months	Severity of urinary incontinence, QOL, cure and improvement rate
Hirakawa et al. (31)	Japan	RCT	EMG-BF plus PFMT	PFMT	23	23	55.3 ± 9.8	58.3 ± 11.2	12 weeks	12 weeks	Severity of urinary incontinence, QOL, pelvic floor muscle strength, cure and improvement rate
Sahin et al. (34)	Turkey	RCT	EMG-BF plus PFMT	PFMT	20	20	61.5 ± 11.2	60 ± 10.6	3 weeks	6 months	Severity of urinary incontinence, QOL, pelvic floor muscle strength

E group, experimental group; C group, control group; EMG-BF, electromyography biofeedback; PFMT, pelvic floor muscle training; NEMS, neuromuscular electrical stimulation; QOL, quality of life; NR, not reported.

Specifically, Goode (29) (USA) randomized 140 participants (70 vs. 70; mean age 66.8 ± 7.0 vs. 66.3 ± 7.5) to EMG-BF plus PFMT or PFMT alone for 8 weeks, with 12-month follow-up, and assessed incontinence severity, QoL, and cure/improvement rate. Liu et al. (32) (China) enrolled 118 participants (58 vs. 60; mean age 50 ± 9.9 vs. 46 ± 11.7) over 12 weeks with 12-week follow-up, assessing incontinence severity and QoL. Özlü et al. (33) (Turkey) *n* = 34(17 vs. 17; mean age: 42.11 ± 8.33 vs. 42.82 ± 6.30), duration: 8weeks + 8week follow-up reported Incontinence severity, QoL, pelvic floor muscle strength, and cure/improvement rate. Wu et al. (35) (China): *n* = 45 randomized (23 vs. 22; mean age 32.2 ± 4.7 vs. 32 ± 5.2), intervention duration: 6 weeks, follow up time: 6 months, evaluating the degree of incontinence and QoL. Bertotto et al. (28) (Brazil) included 31 patients (16 vs. 15; average age 58.4 ± 6.8 vs. 59.3 ± 4.9), with an intervention of 4 weeks and 8 weeks of follow up, assessing the degree of severity of urinary incontinence and PFMT power. Hagen et al. (30) (USA, as indicated in the table), comprised of 600 individuals (300 vs. 300; mean age: 48.2 ± 11.6 vs. 47.3 ± 11.4) for whom an intervention period was 16 weeks and followed by 2 years, reporting on incontinence severity, QoL, and cure/improvement rate. Hirakawa et al. (31) (Japan) enrolled 46 participants (23 vs. 23; mean age 55.3 ± 9.8 vs. 58.3 ± 11.2), delivered a 12-week intervention with 12-week follow-up, and assessed incontinence severity, QoL, pelvic floor muscle strength, and cure/improvement rate. Sahin et al. (34) (Turkey) included 40 participants (20 vs. 20; mean age 61.5 ± 11.2 vs. 60 ± 10.6), delivered a 3-week intervention with 6-month follow-up, and reported incontinence severity, QoL, and pelvic floor muscle strength. Diagnostic criteria varied across studies; most trials used clinician diagnosis with symptom-based definitions of SUI, several required urodynamic confirmation, and one large pragmatic trial included stress UI and mixed UI (stress-predominant) (Table 2).

**Table 2:** Diagnostic criteria and population definitions used in included trials.

Study	Population enrolled	How SUI or eligible UI was defined/confirmed	Mixed population
Hagen et al. (30)	Women with stress UI or mixed UI	Clinically diagnosed stress or mixed UI; mixed UI included when urine leakage was the primary complaint; urgency UI alone excluded	Yes. Included stress UI and mixed UI; outcomes reported for the combined eligible population
Hirakawa et al., (31)	Women with SUI	Diagnosed by a urogynecologist; required leakage episodes >1/week	No (SUI-only)
Bertotto et al. (28)	Postmenopausal women with SUI	Inclusion required complaint of urine loss on exertion (ICIQ-based screening); SUI described per ICS terminology	No (SUI-only)
Özlü et al. (33)	Women with urodynamic SUI	Required urodynamic confirmation of SUI; mild–moderate SUI; also required PFM strength ≥3/5	No (SUI-only)
Sahin et al. (34)	Women diagnosed with SUI	Required SUI diagnosis after urodynamic testing	No (SUI-only)
Wu et al. (35)	Primiparous postpartum women with 2nd-degree laceration	Not a SUI-only trial; outcomes included UDI-6 stress items and LUTS over time (postpartum LUTS cohort)	Yes (non-SUI-specific). Included because stress-related urinary symptoms were measured, but population not defined as SUI
Liu et al. (32)	Women with SUI	Please specify as reported in the trial (e.g., clinician diagnosis/questionnaire/urodynamics)	Pending extraction from full text
Goode (29)	Men with post-prostatectomy incontinence	Not female SUI; diagnosis relates to post-prostatectomy UI with stress/urge/mixed subtypes	Not eligible for female SUI. This citation should be removed from the female SUI RCT table and from the pooled analysis if it was mistakenly included

SUI, stress urinary incontinence; UI, urinary incontinence; PFM, pelvic floor muscle; ICIQ, international consultation on incontinence questionnaire; UDI-6, urogenital distress inventory-short form; LUTS, lower urinary tract symptoms; ICS, international continence society.

### Risk of bias

**Figure 2:**
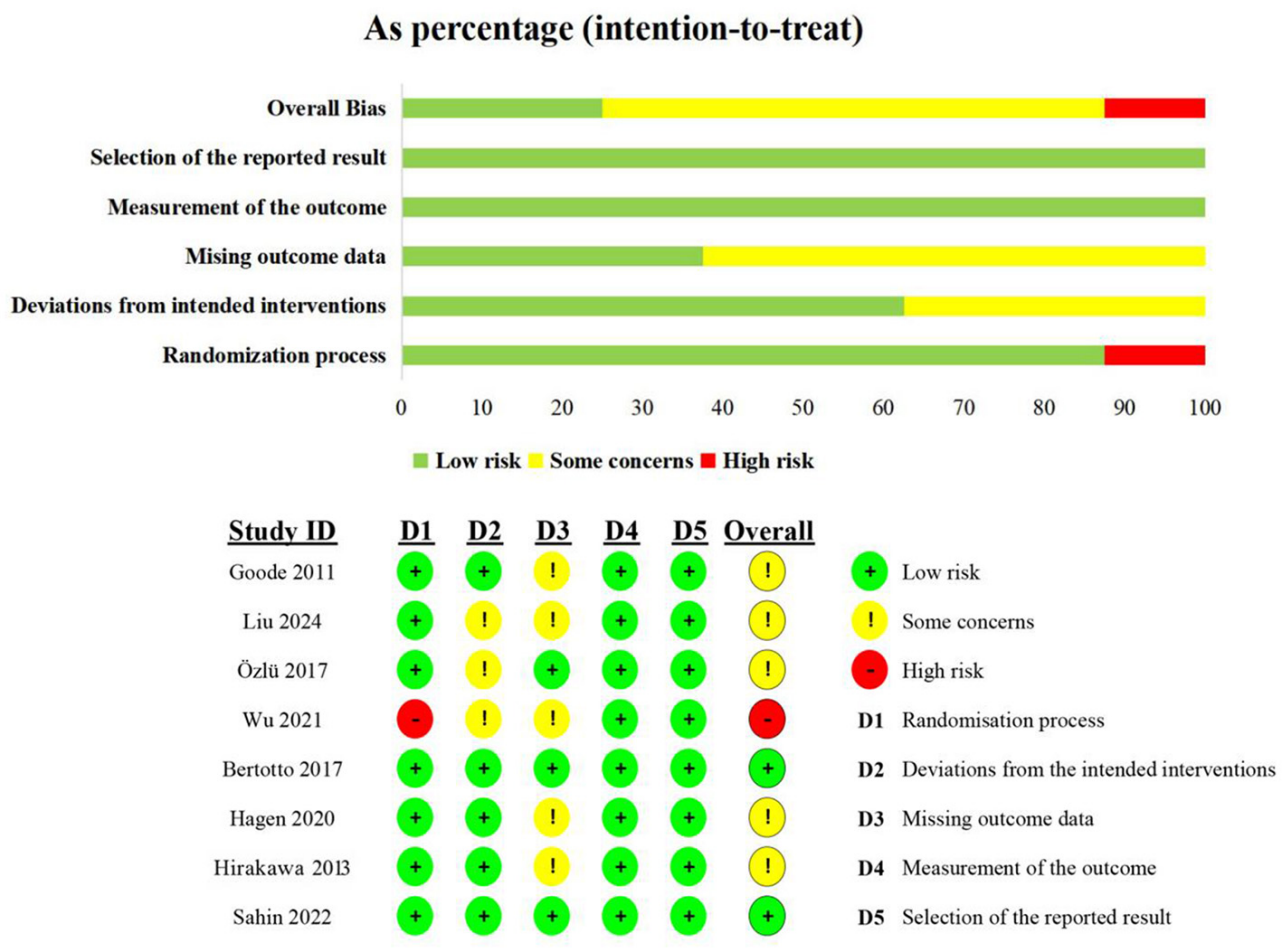
Risk of bias assessment for the included trials, presented as percentages based on intention-to-treat analysis.

Risk of bias was assessed using the RoB 2 tool and summarized as percentages (intention-to-treat) and as study-level judgments across five domains (Figure 2). Overall, most domains were rated at low risk of bias, particularly measurement of outcomes and selection of the reported result, which were consistently judged as low risk across included trials. Concerns were mainly driven by the randomization process and deviations from intended interventions. Specifically, Wu et al. (35) was judged as high risk overall, primarily due to a high-risk judgment in the randomization process. Several trials (28–35) were rated as having some concerns overall, typically reflecting insufficient reporting or potential issues in at least one RoB 2 domain rather than clear evidence of bias. In contrast, Bertotto et al. (28) and Sahin et al. (34) were judged as low risk of bias overall across domains. These findings suggest that, while the included evidence base is generally methodologically acceptable, interpretation of pooled estimates should consider potential bias related to randomization procedures and intervention adherence, and results should be viewed with more caution for studies carrying “some concerns” or “high risk” judgments.

## Effects of interventions

### Incontinence severity

**Figure 3:**
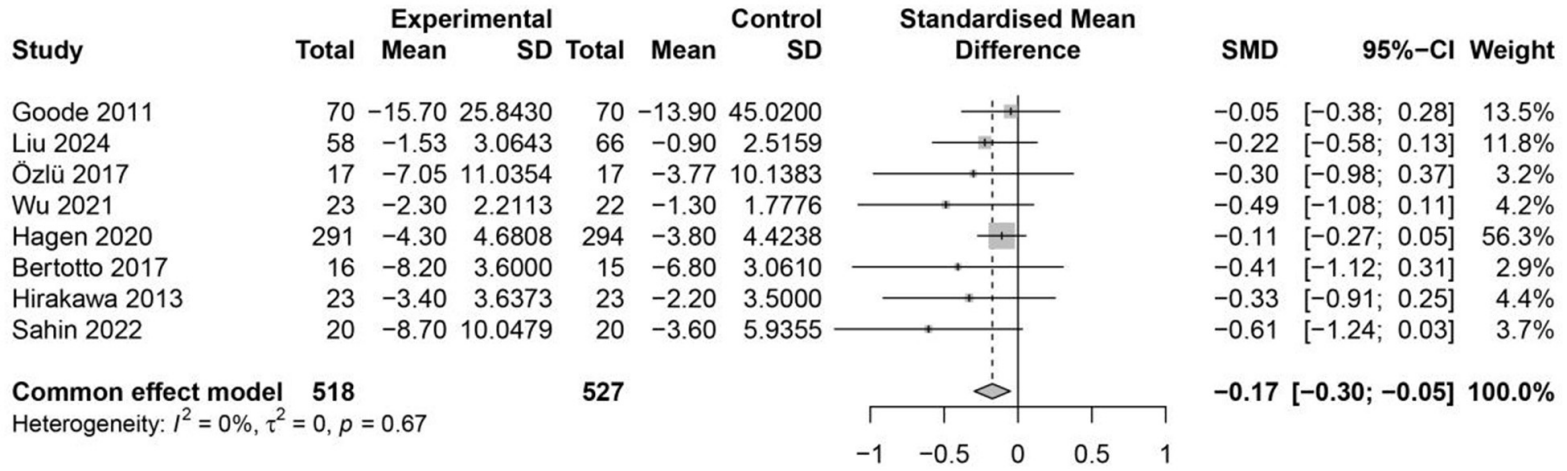
Forest plot of the standardized mean difference for incontinence severity comparing EMG-BF-assisted PFMT vs. control.

Eight trials (28–35) (EMG-BF + PFMT: *n* = 518; PFMT: *n* = 527) contributed to the meta-analysis of incontinence severity. EMG-BF + PFMT produced a small but statistically significant reduction in symptom severity compared with PFMT alone (*SMD* = −0.17, 95% *CI* −0.30 to −0.05; Figure 3). Statistical heterogeneity was negligible (*I*^2^ = 0%, τ^2^ = 0, *p* = 0.67), therefore a fixed-effect model was applied. Across individual studies, effect estimates generally favored EMG-BF + PFMT, with the largest weight contributed by Hagen et al. (30) (56.3%). One study (Wu et al. (35)) showed a larger effect in favor of EMG-BF + PFMT (SMD = −0.49), whereas several studies showed small or null effects (e.g., Goode (29); Liu et al. (32)).

### Pelvic floor muscle strength

**Figure 4:**
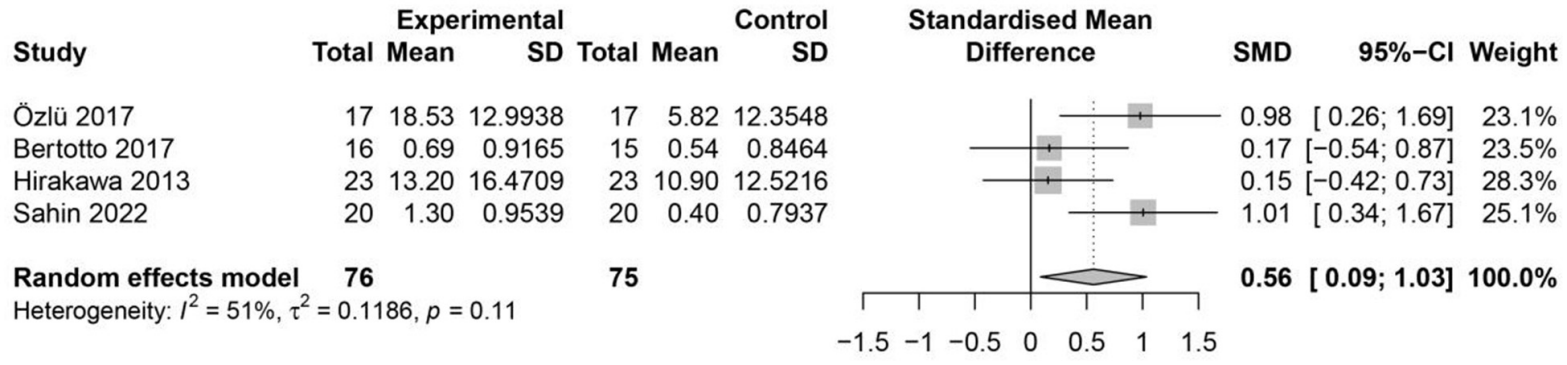
Forest plot of the standardized mean difference for pelvic floor muscle strength comparing EMG-BF-assisted PFMT vs. control.

Four trials (28, 31, 33, 34) contributed data to the meta-analysis of pelvic floor muscle strength (EMG-BF + PFMT: *n* = 76; PFMT: *n* = 75). Overall, EMG biofeedback-assisted PFMT was associated with a moderate improvement in pelvic floor muscle strength compared with PFMT alone (*SMD* = 0.56, 95% *CI* 0.09 to 1.03; Figure 4). Because heterogeneity was moderate (*I*^2^ = 51%, τ^2^ = 0.1186; *p* = 0.11), a random-effects model was applied. At the individual study level, effects generally favored EMG-BF + PFMT, with larger effects observed in Özlü (2017) (*SMD* = 0.98) and Sahin et al. (34) (*SMD* = 1.01), while Bertotto et al. (28) and Hirakawa et al. (31) showed smaller, non-significant differences.

### Quality of life

**Figure 5:**
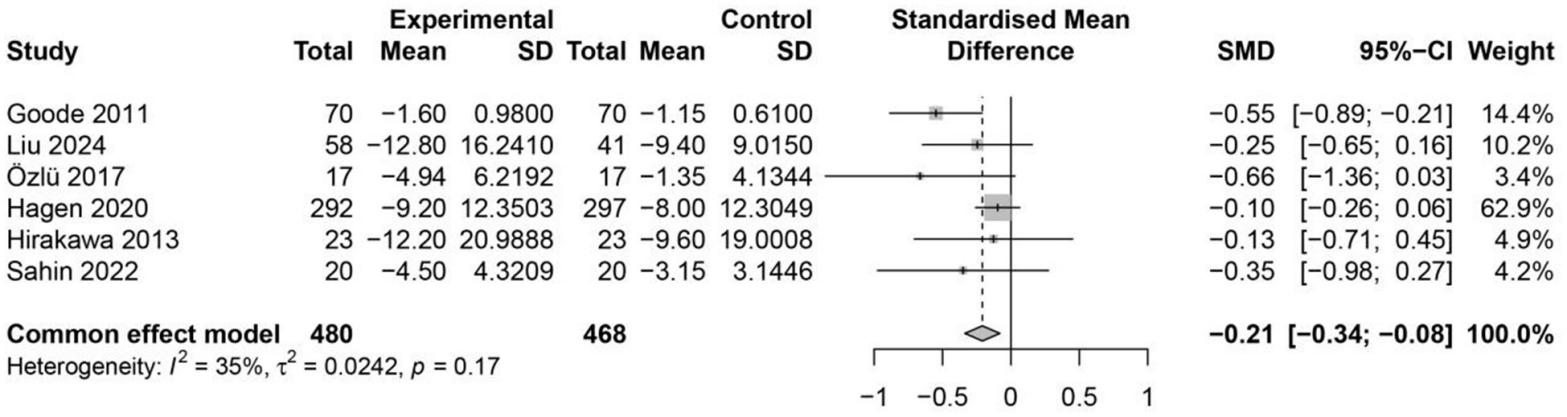
Forest plot of the standardized mean difference for QoL comparing EMG-BF-assisted PFMT vs. control.

Six trials (29–34) contributed data to the meta-analysis of QoL (EMG-BF + PFMT: n = 480; PFMT: *n* = 468). Overall, EMG biofeedback-assisted PFMT was associated with a small but statistically significant improvement in QoL compared with PFMT alone (*SMD* = −0.21, 95% *CI* −0.34 to −0.08; Figure 5). Between-study heterogeneity was low to moderate (*I*^2^ = 35%, τ^2^ = 0.0242; *p* = 0.17), and therefore a fixed-effect model was applied. The pooled estimate was driven largely by the largest trial, Hagen et al. (30) (weight 62.9%), which showed a near-null effect (*SMD* = −0.10). Individual trials showed directionally consistent effects favoring EMG-BF + PFMT, with Goode (29) demonstrating the largest improvement (*SMD* = −0.55), whereas Liu et al. (32) and Hirakawa et al. (31) showed minimal differences.

### Sensitivity analysis

**Figure 6:**
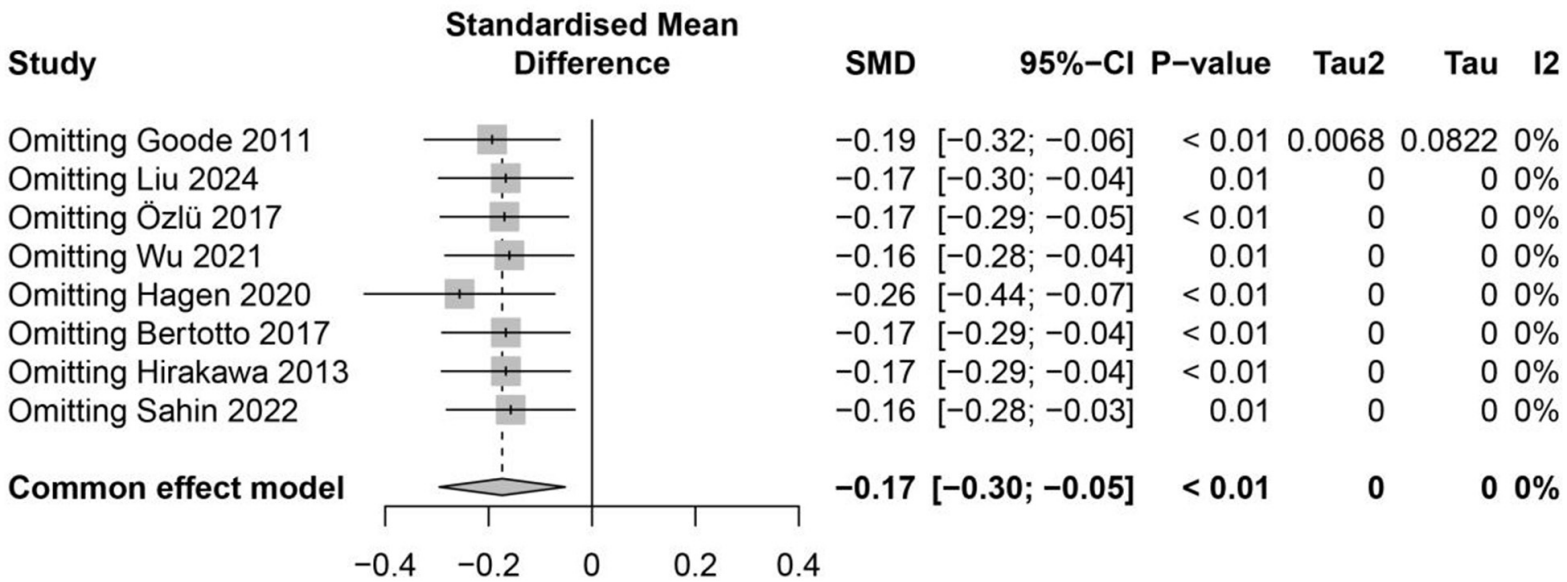
Sensitivity analysis of the pooled SMD for incontinence severity by sequentially omitting each individual study.

A leave-one-out sensitivity analysis was performed for the primary outcome of incontinence severity (Figure 6). Sequential omission of each individual study did not materially change the direction or statistical significance of the pooled estimate, indicating that the overall result was stable and not driven by any single trial (28–35). Across the leave-one-out iterations, pooled effects ranged from *SMD* = −0.16 to *SMD* = −0.26, and all corresponding 95% *CIs* remained below zero (all *p*-values ≤ 0.01). The largest change in magnitude was observed when omitting Hagen et al. (30) (*SMD* = −0.26, 95% *CI* −0.44 to −0.07), but the overall conclusion remained unchanged. Heterogeneity remained negligible across iterations (*I*^2^ = 0%), consistent with the primary analysis.

### Publication bias

**Figure 7:**
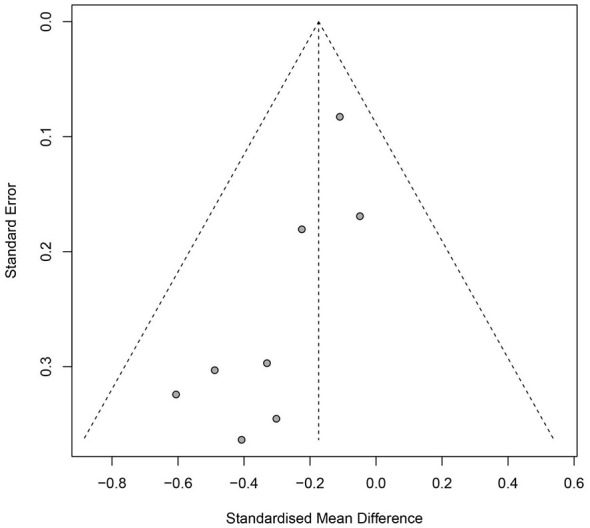
Funnel plot for assessing publication bias in the meta-analysis of EMG-BF-assisted PFMT on incontinence severity.

Potential small-study effects for the incontinence-severity outcome were explored by visual inspection of a funnel plot (Figure 7) and Egger's regression test. The funnel plot appeared asymmetric, and Egger's test suggested asymmetry (*t* = −3.57, df = 6, *p* = 0.0118). Because only eight trials were available, this finding should be interpreted cautiously. In particular, Egger's test is known to have limited reliability when the number of studies is small, and asymmetry in this setting may reflect factors other than publication bias alone. In our dataset, the pattern of one large pragmatic trial with near-null effects alongside several smaller trials showing larger favorable effects may itself contribute to the observed asymmetry. Differences in intervention intensity, supervision, follow-up duration, and outcome instruments may also have played a role. Therefore, these findings are more appropriately interpreted as indicating possible small-study effects rather than definitive publication bias.

## Discussion

### Summary of principal findings

This review pooled data from eight RCTs that compared EMG-biofeedback–assisted PFMT with PFMT alone in women with stress urinary incontinence. Overall, adding EMG-BF produced a small but statistically significant improvement in incontinence severity (*SMD* −0.17) and quality of life (*SMD* −0.21), and a moderate improvement in pelvic floor muscle strength (*SMD* 0.56). Between-study heterogeneity was minimal for incontinence severity, low to moderate for QoL, and moderate for muscle strength.

### Interpretation and clinical relevance of effect sizes

Although the pooled effects for symptom severity and QoL reached statistical significance, their magnitudes were small, suggesting that EMG-BF provides incremental benefit rather than a step-change in clinical response. This pattern is compatible with the established role of PFMT as a first-line conservative intervention for urinary incontinence in women: once PFMT is delivered with adequate training content and supervision, additional gains may be constrained by a ceiling effect (12). In this context, modest average improvements with EMG-BF may still be meaningful for selected individuals, but they do not automatically justify routine implementation across all clinical pathways.

### Why muscle strength improved more than symptoms: a motor-learning explanation

The larger pooled effect for pelvic floor muscle strength relative to symptom severity and QoL is mechanistically plausible. EMG-BF provides immediate external feedback that can help participants identify correct pelvic floor contractions, calibrate effort, and reduce compensatory activation, potentially accelerating neuromuscular learning during PFMT (16, 17). This is clinically relevant because incorrect pelvic floor contraction technique is common, particularly early in training, and can limit real-world PFMT effectiveness (14). However, improved strength does not necessarily translate proportionally into symptom relief, because continence also depends on timing/coordination, reflex pre-contraction, and contextual factors such as psychosocial stress and symptom perception (2, 15). Therefore, strength gains may be more readily detectable than patient-reported symptom change.

### Consistency with prior evidence and the influence of OPAL

Prior evidence syntheses have reported mixed conclusions regarding the additive value of EMG-BF, partly because reviews often combined different incontinence subtypes and heterogeneous intervention packages (21). A central reference point is the OPAL multicentre RCT, which reported no clinically important advantage of adding EMG-BF to PFMT in longer-term outcomes within a structured intervention framework (30). In the present meta-analysis, the overall pooled estimates still favored EMG-BF + PFMT for severity and QoL, but the effect sizes remained small and were heavily informed by the largest study in some outcomes. A reasonable reconciliation is that EMG-BF may offer the greatest marginal value when baseline skill acquisition is poor or when PFMT delivery is less intensive, whereas its benefit may shrink when high-quality PFMT (with supervision and behavioral support) is already in place (12, 30).

### Adherence, delivery intensity, and technology-supported PFMT

Training dose and adherence are likely effect modifiers in this literature. More intensive PFMT protocols can produce larger improvements in symptoms and pelvic floor function (36). In parallel, technology-supported delivery methods—such as apps, reminders, and telehealth—have been investigated as ways to sustain participation and improve training quality. Real-world evaluations of digital health programs show symptomatic improvement in female urinary incontinence users (22), and trials using app-guided PFMT or reminder-based approaches report better self-efficacy and improved electrophysiological parameters (23, 24). These findings highlight that the “value” of EMG-BF may partly depend on whether it meaningfully increases the effective dose of PFMT and correct technique in practice. In settings where adherence barriers dominate, combining feedback with pragmatic support tools (e.g., reminders or teleconsultation) may be more impactful than adding clinic-based technology alone (25). Clinical heterogeneity across trials is also important for interpreting the pooled results. Intervention duration ranged from 3 to 16 weeks, with follow-up extending from 8 weeks to 24 months. Shorter-duration studies may be more likely to capture early gains in motor learning and contraction awareness, which are plausible domains in which EMG biofeedback could exert its greatest effect. By contrast, longer-duration and longer-follow-up trials may provide a more stringent test of whether these early advantages translate into sustained symptom improvement. This distinction may help explain why some smaller, shorter trials reported larger favorable effects, whereas the largest pragmatic trial with longer follow-up found little additional benefit beyond PFMT alone. Mode of delivery and supervision may also modify the observed treatment effect. In trials with greater therapist contact or closely supervised PFMT, the comparator intervention may already provide substantial support for correct muscle activation, adherence, and progression, thereby reducing the incremental benefit attributable to EMG biofeedback itself. Conversely, in more home-based or less intensively supervised settings, EMG biofeedback may play a larger role by reinforcing correct contraction technique and providing external feedback during unsupervised practice. Although the present review was not powered to perform formal subgroup meta-analyses on these factors, the available evidence suggests that intervention duration and supervision intensity are plausible effect modifiers and should be considered when interpreting the average pooled effect.

### Feasibility and acceptability considerations

Implementation choices must also be made with regard to the patients' comfort level, as well as availability of resources: Pelvic floor rehabilitation using invasive devices may cause discomfort or resistance among patients, potentially limiting uptake and persistence (19). Furthermore, there are alternatives that can provide similar gains to EMGBF in some trials, including the use of vaginal cones, suggesting that there are many supporting devices which perhaps serve only as learning assistants and not as separate therapies themselves (34). Thus, selection of an adjunct can be based on availability, cost, clinician skill, and patient preference. An additional issue affecting interpretation is the possibility of small-study effects. For the incontinence-severity outcome, the funnel plot was asymmetric and Egger's regression test was statistically significant (*p* = 0.0118). However, this result should not be overinterpreted as proof of publication bias. First, only eight studies contributed to this analysis, and asymmetry tests such as Egger's regression have limited power and may produce unstable or misleading results when fewer than 10 studies are available. Second, the asymmetry may reflect the structure of the evidence base itself: our meta-analysis included one very large pragmatic trial contributing substantial weight and several relatively small studies that reported larger benefits. Such a pattern can generate apparent small-study effects even in the absence of selective publication. Third, there were between-study differences in PFMT dose, amount of supervision, EMG-BF implementation, follow-up timing, and outcome measurement, all of which may influence effect size estimates. Accordingly, the pooled effect on incontinence severity may be somewhat inflated, and the modest benefit observed should be interpreted conservatively.

### Implications for practice and research

The data suggest that EMG-BF + PFMT could modestly improve the severity of symptoms and QoL but more clearly show an effect in pelvic floor muscle strength. Instead of being used routinely by all patients, EMG-BF seems more suited as an exclusive instrument–especially for women with difficulty recognizing appropriate contractions, or for whom extra structure and support are required in the initial stages of learning (14, 17). Future RCTs must (i) match contact time and PFMT dose between arms in order to more clearly separate out the impact of EMGBF, (ii) *a priori* define subgroups by baseline contraction ability and symptoms severity, and (iii) include longer follow-up with standardized outcome sets, and adherence measures in order to shed light on durability and mechanisms of benefit (12, 30). Clinical heterogeneity should be considered when interpreting these findings. The included trials varied substantially in intervention duration (3–16 weeks), follow-up length, and delivery format. Shorter studies may be more likely to detect early benefits of EMG biofeedback on motor learning, contraction awareness, and initial strength gains, whereas longer-duration studies with extended follow-up provide a better estimate of whether these advantages are sustained over time. Similarly, the degree of supervision may influence the incremental effect of EMG biofeedback. When PFMT is delivered with close therapist supervision, the control intervention may already optimize technique and adherence, leaving less room for additional benefit from biofeedback. In contrast, in more home-based or less intensively supervised programs, EMG biofeedback may offer greater support for correct contraction and self-monitoring. Because the number of included studies was limited and reporting was not sufficiently uniform, these factors could not be examined in robust quantitative subgroup analyses and should therefore be viewed as hypothesis-generating.

## Conclusion

This meta-analysis of eight RCTs suggests that adding EMG-BF to PFMT yields small improvements in incontinence severity and QoL, and a moderate improvement in pelvic floor muscle strength compared with PFMT alone. Given the limited magnitude of symptom and QoL effects, the presence of possible small-study effects, and the likelihood that training dose and supervision differ across trials, EMG-BF should not be viewed as a universal add-on for all women with SUI. Instead, it appears most appropriate as a selective adjunct, especially for individuals who struggle to perform an isolated pelvic floor contraction or who need additional feedback during early skill acquisition. Future trials should better match therapist contact time and exercise dose between groups, prespecify clinically relevant subgroups (e.g., poor baseline contraction ability), and adopt standardized outcome sets with longer follow-up to clarify durability and identify the patients most likely to benefit.

## Data Availability

The original contributions presented in the study are included in the article/supplementary material, further inquiries can be directed to the corresponding author/s.
